# Ferritin, Serum Iron and Hemoglobin as Acute Phase Reactants in Laparoscopic and Open Surgery of Cholecystectomy: An Observational Prospective Study

**DOI:** 10.3390/pathophysiology29040045

**Published:** 2022-10-11

**Authors:** Cristina Vila Zárate, Candelaria Martín González, Ruimán José González Álvarez, Iván Soto Darias, Beatriz Díaz Pérez, Pedro Abreu González, Vicente Medina Arana, Antonio Martínez Riera

**Affiliations:** 1 Emergency Surgery Unit, General Surgery Department, Hospital Universitario Nuestra Señora de la Candelaria (HUNSC), Carretera General del Rosario 145, 38010 Santa Cruz de Tenerife, Santa Cruz de Tenerife, Spain; 2 Intern Medicine Department, Hospital Universitario de Canarias (HUC), Carretera Ofra S/N, 38320 San Cristóbal de La Laguna, Santa Cruz de Tenerife, Spain; 3 Urology Department, Hospital Universitario de Canarias (HUC), Carretera Ofra S/N, 38320 San Cristóbal de La Laguna, Santa Cruz de Tenerife, Spain; 4 General Surgery Department, Hospital Universitario Materno-Infantil Insular de Las Palmas de Gran Canaria, Avenida Marítima del Sur, S/N, 35016 Las Palmas de Gran Canaria, Las Palmas, Spain; 5 General Surgery Department, Hospital Universitario de Canarias (HUC), Carretera Ofra S/N, 38320 San Cristóbal de La Laguna, Santa Cruz de Tenerife, Spain; 6 Microbiology Department, Hospital Universitario de Canarias (HUC), Carretera Ofra S/N, 38320 San Cristóbal de La Laguna, Santa Cruz de Tenerife, Spain

**Keywords:** inflammatory response, acute phase reactants, cytokines, ferritin, N/L ratio, interleukin 6, CRP, hemoglobin

## Abstract

Cytokines are expressed by various cells after several stimuli such as surgical tissue damage, producing a systemic inflammatory response (SIR). C-reactive protein (CRP) is used extensively in clinical practice after operative injury, but proinflammatory cytokines, iron status, albumin, neutrophil-to-lymphocyte (N/L) ratio and hemoglobin, as acute phase reactants, have been poorly documented. This study aims to show how they behave after surgery, comparing laparoscopic (LC) versus open cholecystectomy (OC). In total, 55 patients were included in a prospective non-randomized form to undergo a cholecystectomy: 8 patients OC (50% females) and 47 patients LC (68% females). Before (A1) and 24 h after surgery (A2), blood samples were taken for an ordinary analysis and IL6, IL8 and TNFα determination. There were no differences between LC and OC groups concerning age, CRP, IL6 and TNFα at day A1. In the LC group at day A2, CRP, IL6, IL8, TNF, ferritin, leukocytes and N/L ratio increased; hemoglobin, lymphocytes, prothrombin and albumin decreased (*p* < 0.05). In the OC group at day A2, only IL6 (*p* < 0,07), ferritin, leukocytes, N/L ratio and CRP (*p* < 0.05) increased; serum iron, hemoglobin, lymphocytes and albumin (*p* < 0.05) decreased. At day A2, OC vs. LC group, higher values were observed in IL6, ferritin and CRP (*p* ≤ 0.05), and lesser values were observed in serum iron and prothrombin (*p* < 0.05). In conclusion, classic markers of inflammation are altered after surgery, in a milder way in laparoscopic surgery. Ferritin can be used as an inflammatory marker, as has been described in COVID-19 infection.

## 1. Introduction

Cytokines are expressed and secreted by various cells after stimuli such as pathogen invasions or tissue damage associated with surgical procedures, such as the manipulation and damage of the peritoneum during laparotomy. These cytokines produce a systemic inflammatory response (SIR), the so-called acute phase response (APR), which is nonspecific and correspond to the severity of the aggression [1]. The intensity of the APR determines the prognosis; if excessive, this response can be harmful and may lead to complications after surgery [2], or even to death.

The intensity of this inflammatory process may be assessed using multiple acute phase reactants (for instance, serum albumin, C-reactive protein (CRP), α2 macroglobulin and ferritin, among many others) that may estimate the magnitude of the surgical trauma and also bear prognostic value.

CRP is routinely measured and widely used worldwide. It may be useful in monitoring SIRS after elective surgery [2,3]. In contrast, interleukin (IL)-6, IL-8 or tumor necrosis factor (TNF) α, are used, usually, only in research. IL-8 activity correlates with IL-6 levels after injury or surgical trauma, modulates the number of circulating granulocytes and attracts them to the site of injury [4,5].

Iron metabolism-related variables (ferritin, serum iron); the ratio of neutrophil to lymphocyte counts (in absolute and/or relative percentage values), termed neutrophil–lymphocyte stress factor in the clinical setting [6]; and hemoglobin have not been well studied in surgery-related APR. Iron metabolism is strongly influenced by the primary mediators of the APR, namely TNF-α and IL-1 [7,8], that lead to increased levels of ferritin, a molecule that has recently gained interest as a very reliable marker of intense cytokine release in patients affected by COVID-19 infection. Laparoscopic cholecystectomy (LC) is a so-called “minimally invasive” surgical procedure. It causes less tissue trauma than open surgery and seems to preserve the immune function better. Based on these considerations, the present prospective non-randomized study aims to describe the analytical behavior of indices of albumin, hemoglobin and the ratio of neutrophil to lymphocyte counts (in %), as well as CRP and proinflammatory cytokines, in patients after laparoscopic cholecystectomy (LC) compared to patients undergoing open cholecystectomy (OC). A secondary aim was to analyze iron status (serum ferritin and serum iron concentrations) as an inflammatory reactant during surgical aggression as has been described in COVID-19 patients.

## 2. Materials and Methods

Recruitment was performed at Hospital Universitario de Canarias, a third-level public hospital with 822 beds, between June 2016 and July 2018 in a prospective non-randomized form among patients who underwent a cholecystectomy. The study protocol was approved by the local ethical committee of our hospital (code 2016_01) and conforms to the ethical guidelines of the 1975 Declaration of Helsinki. All patients and controls gave their written informed consent.

The patients included were adults who had acute cholecystitis in the past, treated with antibiotics for one week, at least 6 weeks before the elective surgery. Patients with acute cholecystitis at the time of the surgery, another concurrent infection, a neoplasm at the time of the surgery or no completed follow-up were excluded. In total, 55 patients were included: laparoscopy (LC), 47 patients (68% females), and open (OC), 8 patients (50% females). The indication for the open approach was anesthetic risk, and in this group, we included laparoscopy conversions (4 cases) in the analysis. Both procedures were performed by members of the research team. The median age was 64.0 years (IQR 21) with no differences between sexes or between the type of intervention.

Before (A1) and 24 h after surgery (A2), whole blood samples were taken at 8:00 a.m. in fasting conditions and were sent to HUC Laboratory Service for routine automatic laboratory evaluation (blood count, ferritin, serum iron, albumin levels and prothrombin) and immediately frozen at −20 °C in the HUC biobank until assay of IL-6, IL-8 and TNF-α.

IL-6 serum levels were determined by ELISA (Bender Med Systems, Vienna, Austria). Inter-assay and intra-assay coefficients of variation were 6.3% and 3.8%, respectively. The limit of detection was established at 0.047 pg/mL. IL-8 serum levels were determined by ELISA (Bender Med Systems, Vienna, Austria). Inter-assay and intra-assay coefficients of variation were 6.1% and 3.9%, respectively. The limit of detection was established at 0.038 pg/mL. Serum TNF-α was determined by immunometric chemiluminescent assay (intra-assay variation coefficient ranging from 4 to 6.5%, inter-assay variation coefficient ranging from 2.6 to 3.6%, recovery 92–112%; Diagnostic Products Corporation, Los Angeles, CA, USA). Therefore, we have followed the same method followed in other studies [9].

### Statistical Analysis

IBM SPSS Statistics V25.0 software (La Laguna University, Santa Cruz de Tenerife, Spain) was used for statistical analysis. The Kolmogorov–Smirnov test was used to test for normal distribution, a condition not fulfilled by several variables. Therefore, non-parametric tests, such as Mann−Whitney’s U-test for independent samples or Wilcoxon signed-rank test to compare related samples, were used to analyze differences in these parameters between groups. Student’s *t*-test, or paired *t*-test, and Pearson’s correlation analysis were used for the variables with a normal distribution, whereas Spearman’s rho was utilized in the case of non-parametric variables. A *p* value  <  0.05 indicates statistical significance.

## 3. Results

There were no differences between LC and OC groups concerning age, sex, hemoglobin, leukocytes, neutrophils, lymphocytes, serum iron, ferritin, IL-6, Il-8, MDA, CRP, albumin, IL-6 and TNF-α at day 0 (A1), but prothrombin was lower in the OC group (prothrombin LC 99% IQR 9 versus OC 78% IQR 13, Z = −2,8, *p* < 0.005).

**Figure 1:**
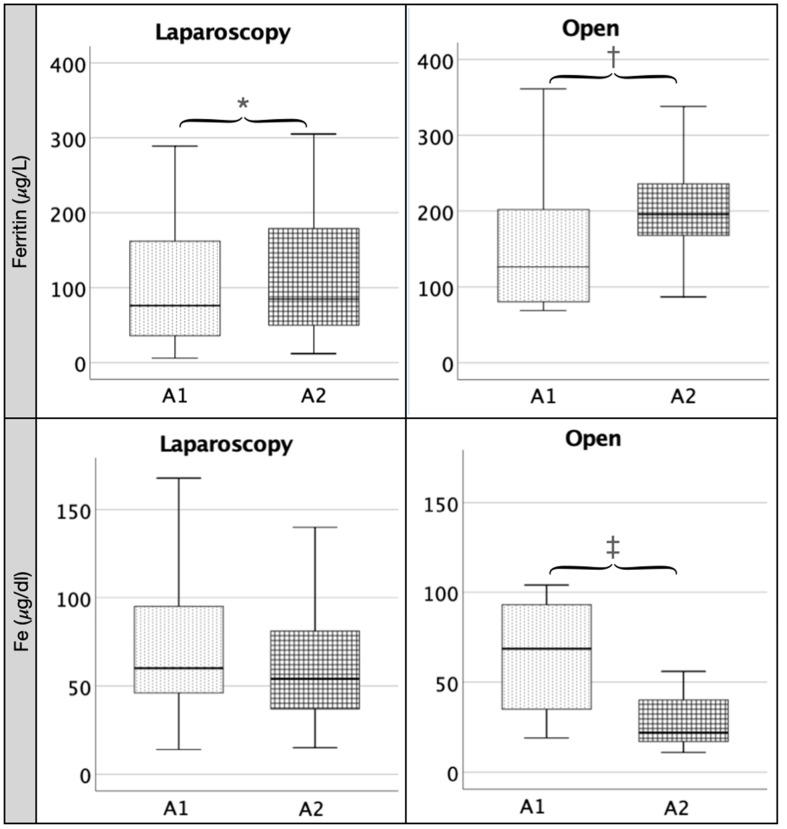
Comparison between A1 and A2 in laparoscopic (LC) and open cholecystectomy (LC): ferritin and Fe. Legend: Both in the laparoscopy and the open group, there is a significant increase in ferritin between baseline (A1) and 24 h (A2) values (∗ = 0.00; † = 0.03). In the open group, there is a significant decrease in Fe between A1 and A2 values (‡ = 0.01).

**Figure 2:**
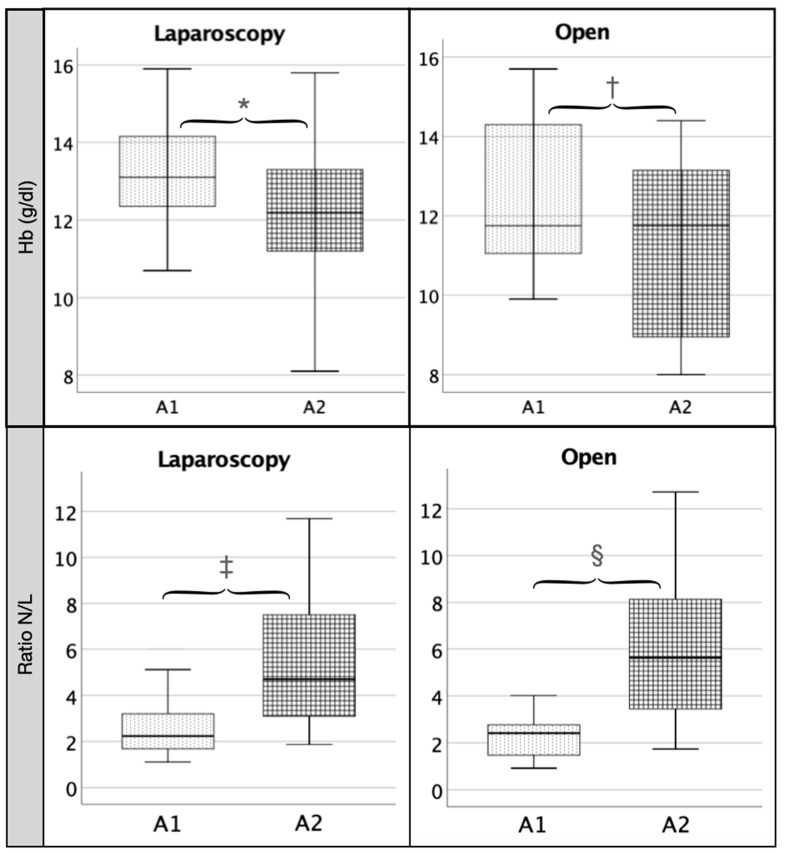
Comparison between A1 and A2 in laparoscopic (LC) and open cholecystectomy (OC) of hemoglobin and N/L ratio. Legend: Both in the laparoscopy and the open group, there is a significant decrease in Hb (∗ = 0.00; † = 0.03) and a significant increase in the N/L ratio (‡ = 0.001; § = 0.02) between baseline (A1) and 24 h (A2) values.

When we analyzed the changes in the measured data before and after surgery within each group (LC and OC), we found that in the LC group (Table 1), there was a significant increase between values at baseline (A1) and at 24 h (A2) of IL-6 (*p* < 0.001), IL-8 (*p* < 0.001), ferritin (*p* < 0.001) (Figure 1), leukocytes (*p* < 0.001), neutrophil (N) percentage (*p* < 0.001) and N/L ratio (*p* < 0.001) (Figure 2) and CRP (*p* < 0.001) and a significant decrease in TNF (*p* < 0.001), Hb (*p* < 0.001) (Figure 1), lymphocyte percentage (*p* < 0.001), albumin (*p* < 0.001) and prothrombin (*p* < 0.001). We also found a non-significant decrease in serum iron between A1 and A2 (Figure 1).

In the OC group, paired-samples comparisons (Table 2) showed a significant increase in ferritin (*p* < 0.03) (Figure 2), leukocyte (*p* < 0.01) and neutrophil percentages (*p* < 0.01), N/L ratio (*p* < 0.02) (Figure 1) and CRP (*p* < 0.02). Both IL-8 and IL-6 showed an increase in their values without reaching significance (*p* = 0.07 and *p* = 0.06, respectively). We found a significant decrease (*p* < 0.05) in serum iron (*p* < 0.01) (Figure 2), Hb (*p* < 0.03) (Figure 1), lymphocytes (*p* < 0.01) and albumin (*p* < 0.01). TNF decreased at 24 h in a non-significant fashion.

**Figure 3:**
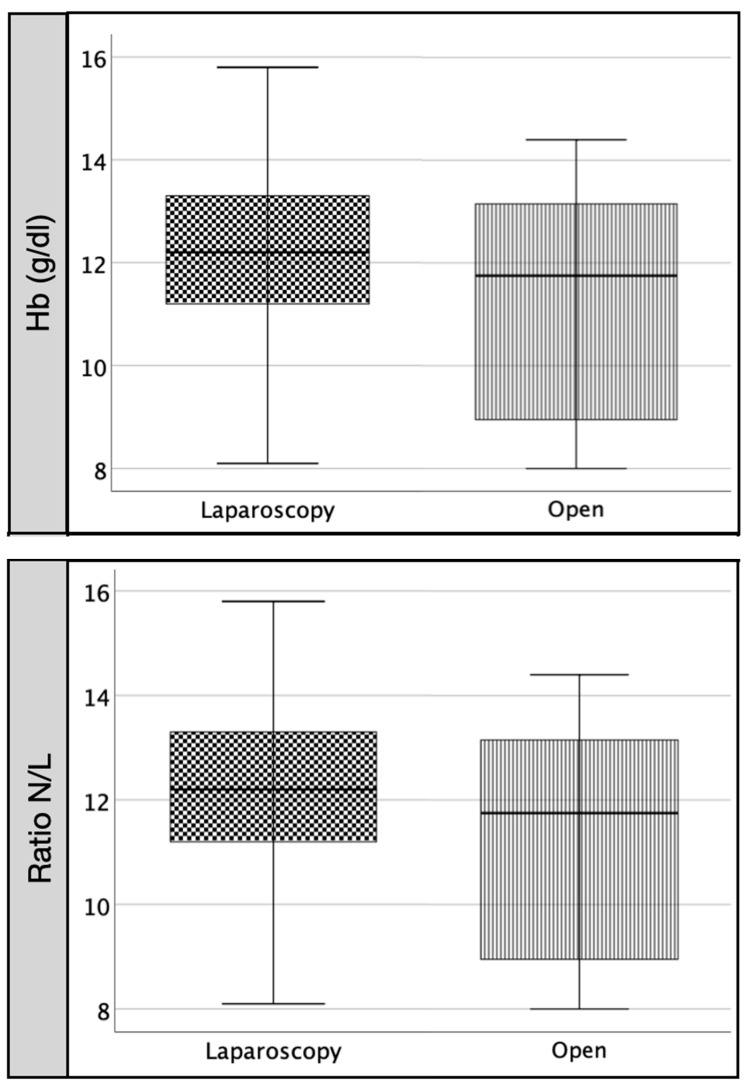
Comparison between LC and OC at postoperative day 1 (A2): hemoglobin and N/L ratio. Legend: Comparing open (OC) versus laparoscopy (LC) group 24 h after surgery (A2), we do not find differences in Hb, but there in OC there is a non-significant increase in N/L ratio.

**Figure 4:**
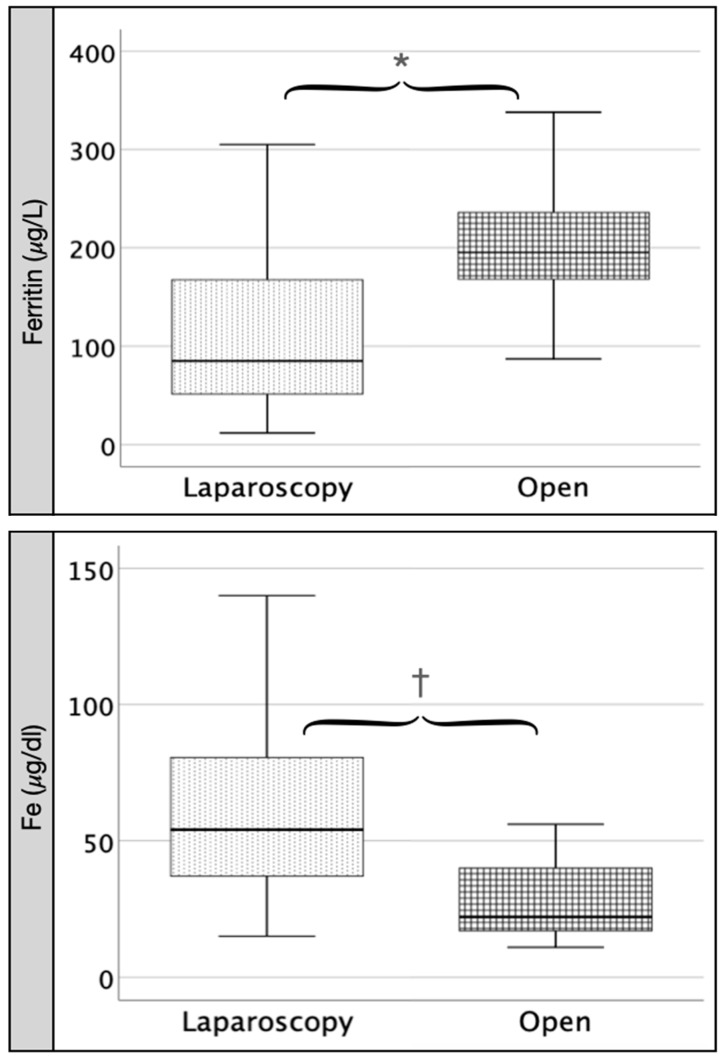
Comparison between LC and OC at postoperative day 1 (A2): ferritin and Fe. Legend: Comparing open (OC) versus laparoscopy (LC) group 24 h after surgery (A2), we find a significant increase in ferritin in OC (∗ = 0.01) and a significant decrease in Fe in OC († = 0.01).

However, when we compared the same parameters between both groups 24 h after surgery (A2), we found significantly higher values in the OC group than in the LC group for IL-6 (*p* < 0.05), ferritin (*p* < 0.01) and CRP (*p* < 0.001) and not significantly higher values for IL-8, TNF, leukocytes, neutrophils and N/L ratio (Table 3). We found significantly lower values (*p* < 0.05) in OC than in LC for serum iron (*p* < 0.01) and prothrombin (*p* < 0.01). We found non-significant differences in the remaining included variables (Figure 3 and Figure 4). There was positive correlation between IL-6 and CRP (rho = 0.64, *p* < 0.001) and inverse correlation between IL-6 and albumin (rho = −0.45, *p* < 0.003), lymphocytes (rho = −0.32, *p* < 0.03), serum iron (rho = −0.37, *p* < 0.01) and prothrombin (rho = −0.36, *p* < 0.01).

Complications were one case of pneumonia and two wound seromas; no correlations between them and inflammatory parameters were found. We had no mortality in our series.

## 4. Discussion

There is no doubt that surgical trauma, even after minimally invasive surgical procedures, such as laparoscopy, induces an inflammatory and metabolic response. Furthermore, this response depends on the severity of tissue injury [4]. The laparoscopic technique was found to be both safe and effective and better than open surgery, especially regarding postoperative morbidity, pain and time to recovery [10]. In the era of laparoscopy as the gold standard procedure for cholecystectomy [11], there is a conversion rate to open surgery of 2–10%. Conversion to open surgery in real life is chosen in cases of heavily inflamed situations when anatomy is unclear and safe laparoscopic surgery does not seem to be feasible. Open cholecystectomy changed from being the standard procedure to a rarely performed rescue maneuver [12].

Chmielecki et al. (2012) studied cirrhotic patients who underwent cholecystectomy. In their study, before the intervention, OC was already chosen in 11.8% of the cases, but a further 15.9% of the patients were converted to OC. Therefore, in pluripathological patients, the global need for OC can be as high as 25% [13].

Our overall OC rate, including conversion, was 14.5%: the eight patients included for OC. In six cases OC was previously decided, based on impaired clinical situation, and further cases were converted to open due to problems in performing the LC. However, despite the low number of patients subjected to OC, the differences between both groups when assessing postoperative inflammation are statistically very significant, strongly validating our results.

When assessing APR by IL-6 and CRP levels, we found that both were elevated at 24 h after the intervention. Our results are similar to those obtained by other authors [14,15,16], including a meta-analysis of Watt et al. (2015) [17], regarding both the elevation of IL-6 and CRP at 24 h and the significantly higher values in OC patients. In contrast to IL-6, however, CRP is routinely measured in clinical laboratories worldwide and widely used in clinical practice; therefore, it may be useful in the detection and evolutive evaluation of the SIR after an elective operation.

The analysis of iron metabolism has gained relevance after the COVID-19 pandemic. Patients with clumsy evolution had raised levels of ferritin as a result of an important innate immune reaction [18]. Many aspects of the fundamental biology of serum ferritin remain surprisingly unclear. Liver iron and several proinflammatory cytokines induce ferritin synthesis [7]. Ferritin, the structure of which resembles a hollow cage, sequesters iron molecules in its core and therefore protects the cells from iron-mediated oxidative damage derived from the formation of highly reactive radicals through Haber–Weiss or Fenton chemistry, storing iron in a non-toxic form. However, increased ferritin levels also represent an important host defense mechanism that sequesters iron, hampering bacterial growth, and protects immune cell function, modulating the immune response by suppressing lymphocyte blastogenesis and myelopoiesis [8].

When we analyzed iron metabolism in our study, we found a decrease in serum iron and a significant increase in ferritin, more intense in patients in the OC group, consistent with the previously mentioned findings, but not described in either open or laparoscopic surgery. In healthy patients undergoing bronchoscopy [19], ferritin determined 24 h after bronchoscopy increases by 25% with a decrease in serum iron.

We did find significant correlations between IL-6 and CPR with serum iron (inverse), but not with ferritin, fully in accordance with the behavior of iron metabolism in APR. There is no doubt, therefore, that in the first 24 h there is a decrease in circulating iron and an elevation of ferritin that are more marked in open surgery as an expression of a more intense inflammatory reaction due to tissue injury [20].

We found a significant decrease in hemoglobin at 24 h after surgery in both types of interventions (LC and OC) with no difference between them. We only found a correlation between hemoglobin and ferritin. However, serum iron correlates with hemoglobin, ferritin and inflammatory variables such as IL-6 and CRP, results that are probably in relation to the effect of hepcidin, another acute phase reactant. During acute inflammation, levels of hepcidin increase, leading to a decrease in serum iron levels [21]. Inflammation causes a reduction in serum iron levels because the increase in hepcidin reduces iron transport out of cells. Blood loss needing transfusion is very low in LC (between 0.48 and 1.3%), although significantly higher in OC (13%) [21], and therefore it should not be considered as the main factor responsible for the decrease in hemoglobin [22].

The physiologic response of circulating leukocytes to surgical stress is characterized by the onset of marked neutrophilia and significant lymphocytopenia following surgery, although such changes in these white cells after an elective operative injury have been poorly documented [23]. The N/L ratio is an easily measurable parameter that may correlate with the severity of the injury. It has been suggested that the N/L ratio should be routinely used for monitoring inflammation after surgery [6,24].

Hypoalbuminemia is associated with inflammation due to increased capillary permeability that favors transcapillary leakage of serum albumin. Decreased serum albumin levels are independent predictors of short-term complications [25], and they are also associated with decreased life expectancy [26]. In our study, both groups experienced a significant decrease in albumin, more marked in the OC group, an expression of inflammation after the surgery, but we failed to find significant differences between LC and OC.

In conclusion: these classical markers (ferritin, albumin, iron, serum hemoglobin and the N/L ratio) of inflammation are altered after surgery. We have documented that laparoscopic surgery, despite its minimally invasive nature, modifies iron metabolism in a milder way, with an increase in ferritin and a decrease in hemoglobin and serum iron, and also causes an early increase in the N/L ratio, in parallel with the elevation of IL-6 and CRP, all 24 h after the procedure. The present data seem to support the view that laparoscopic surgery results in a lower inflammatory response than open surgery. The increased value of ferritin can be used as a simple marker of inflammation, as occurs in COVID-19 infection. It would be useful to study the prognostic value of these markers as predictors of both complications and mortality; they might allow us to monitor patients’ recovery after surgery and, therefore, identify patients at increased risk of developing complications based on the intensity of the SIR [23].

## Figures and Tables

**Table 1:** Comparison between A1 and A2 in laparoscopic cholecystectomy (LC).

	A1	A2	Statistic	*p*
IL-6 pg/mL *	6.9 (5.6)	22.2 (17.7)	3.9	0.001
IL-8 pg/mL *	9.4 (9.15)	12.48 (17.48)	−2.59	0.00
TNF-∝ pg/mL †	24.45 (12.96)	19.72 (12.19)	−3.16	0.00
MDA μmol/L *	2.43 (2.84)	2.38 (1.98)	−1.45	0.14
Ferritin μg/L *	81 (143)	85 (129)	−3.26	0.00
Iron serum µg/dL †	72.39 (40.61)	65.25 (40.99)	−0.94	0.34
Hemoglobin g/dL †	13.14 (1.41)	12.23 (1.72)	−5.12	0.00
Prothrombin % *	99 (9)	91 (21.25)	−3.94	0.00
Leukocytes cells/mm^3^ †	7232.01 (2454.87)	9386.3 (3540.53)	−4.73	0.00
Neutrophils % †	61.71 (9.24)	74.21 (8.87)	−5.36	0.00
Lymphocytes % †	26.63 (8.4)	17.01 (7.06)	−5.28	0.00
N/L ratio *	2.24 (1.22)	4.70 (4.55)	−5.3	0.001
CRP mg/L *	2.6 (8.7)	15.7 (30.25)	−5.49	0.00
Albumin g/dL †	4.28 (0.38)	3.79 (0.39)	−5.38	0.00

Statistic: (*) interquartile range and Z; (†) standard deviation and t.

**Table 2:** Comparison between A1 and A2 in open cholecystectomy (OC).

	A1	A2	Statistic	*p*
IL-6 pg/mL *	16.6 (4.7)	48.4 (23.5)	1.8	0.07
IL-8 pg/mL *	13.92 (20.71)	17.17 (15,47)	−0.7	0.48
TNF-∝ pg/mL †	26.60 (14.53)	19.95 (9.1)	−1.4	0.16
MDA μmol/L *	2.67 (2.44)	2.08 (1.06)	−1.4	0.16
Ferritin μg/L *	126 (133.25)	195.5 (86.5)	−2.1	0.03
Iron serum µg/dL †	64.5 (32.28)	28.12 (17.5)	−2.52	0.01
Hemoglobin g/dL †	12.47 (2.06)	11.26 (2.46)	−2.1	0.03
Prothrombin % *	78 (13)	76.5 (22)	−1.36	0.17
Leukocytes cells/mm^3^ †	7245 (2047.9)	11,433.75 (2415.89)	−2.38	0.01
Neutrophils % †	58.4 (10.8)	76.27 (9.52)	−2.52	0.01
Lymphocytes % †	29.27 (9.96)	16.37 (8.64)	−2.52	0.01
N/L ratio *	2,42 (1.62)	5.60 (5.28)	−2521	0.02
CRP mg/L *	3.45 (43.97)	70.2 (47.7)	−2.24	0.02
Albumin g/dL †	3.95 (0.71)	3.42 (0.66)	−2.52	0.01

Statistic: (*) interquartile range and Z; (†) standard deviation and t.

**Table 3:** Comparison between LC and OC at postoperative day 1 (A2).

	LC	OC	Statistic	*p*
IL-6 pg/mL *	22.18 (28.1)	48.43 (52.7)	101	0.057
IL-8 pg/mL *	12.48 (17.48)	17.17 (15.47)	124	0.21
TNF-∝ pg/mL †	19.72 (12.19)	19.95 (9.10)	−0.04	0.96
MDA μmol/L *	2.38 (1.98)	2.08 (1.06)	153.5	0.56
Ferritin μg/L *	85 (129)	195.5 (86.5)	74.5	0.01
Iron serum µg/dL †	65.25 (40.99)	28.12 (17.5)	2.5	0.01
Hemoglobin g/dL †	12.23 (1.72)	11.26 (2.46)	1.35	0.96
Prothrombin % *	91 (21,25)	76.5 (22)	72.5	0.01
Leukocytes cells/mm^3^ †	9386.3 (3540.53)	11,433.75 (2415.89)	−1.56	0.12
Neutrophils % †	74.21 (8.87)	76.27 (9.52)	−0.59	0.55
Lymphocytes % †	17.01 (7.06)	16.37 (8.64)	0.22	0.82
N/L ratio *	5.424 (2.8257)	6.1103 (3.5481)	−0,6	0.54
CRP mg/L *	15.7 (30.25)	70.20 (47.7)	178	0.00
Albumin g/dL †	3.79 (0.39)	3.42 (0.66)	1.52	0.16

Statistic: (*) interquartile range and U; (†) standard deviation and t.

## Data Availability

The datasets used and/or analyzed during the current study are available from the corresponding author on reasonable request.

## Abbreviations

SIR: systemic inflammatory response; CRP: C-reactive protein; LC: laparoscopic cholecystectomy; OC: open cholecystectomy; APR: acute phase response; TNFα: tumor necrosis factor α; IL: interleukin; N/L ratio: neutrophil/lymphocyte ratio.

## References

[1] Gabay C., Kushner I. (1999). Acute-Phase Proteins and Other Systemic Responses to Inflammation. N. Engl. J. Med..

[2] Migita K., Matsumoto S., Wakatsuki K., Kunishige T., Nakade H., Miyao S., Sho M. (2019). Postoperative Serum C-Reactive Protein Level Predicts Long-term Outcomes in Stage I Gastric Cancer. J. Surg. Res..

[3] Nurmi A.M., Mustonen H.K., Stenman U.-H., Seppänen H.E., Haglund C.H. (2021). Combining CRP and CA19-9 in a novel prognostic score in pancreatic ductal adenocarcinoma. Sci. Rep..

[4] Krog A.H., Sahba M., Pettersen E.M., Sandven I., Thorsby P.M., Jørgensen J.J., Sundhagen J.O., Kazmi S.S. (2016). Comparison of the acute-phase response after laparoscopic versus open aortobifemoral bypass surgery: A substudy of a randomized controlled trial. Vasc. Health Risk Manag..

[5] Tsamis D., Theodoropoulos G., Stamopoulos P., Siakavellas S., Delistathi T., Michalopoulos N.V., Zografos G.C. (2011). Systemic inflammatory response after laparoscopic and conventional colectomy for cancer: A matched case–control study. Surg. Endosc..

[6] Zahorec R. (2001). Ratio of neutrophil to lymphocyte counts--rapid and simple parameter of systemic inflammation and stress in critically ill. Bratisl. Lek. Listy.

[7] Gao G., Li J., Zhang Y., Chang Y.-Z. (2019). Cellular Iron Metabolism and Regulation. Adv. Exp. Med. Biol..

[8] Kernan K.F., Carcillo J.A. (2017). Hyperferritinemia and inflammation. Int. Immunol..

[9] Martín-González C., Martín-Ponce E., Fernández-Rodríguez C., Sánchez-Pérez M.J., Rodríguez-Gaspar M., De-La-Vega-Prieto M.J., Martínez-Riera A., González-Reimers E. (2019). Transforming Growth Factor Beta 1 and Vascular Risk in Alcoholics. Alcohol Alcohol..

[10] Frazee R.C., Roberts J.W., Okeson G.C., Symmonds R.E., Snyder S.K., Hendricks J.C., Smith R.W. (1991). Open versus laparoscopic cholecystectomy. A comparison of post-operative pulmonary function. Ann. Surg..

[11] Visser B.C., Parks R.W., Garden O.J. (2008). Open cholecystectomy in the laparoendoscopic era. Am. J. Surg..

[12] Nebiker C.A., Mechera R., Rosenthal R., Thommen S., Marti W.R., von Holzen U., Oertli D., Vogelbach P. (2015). Residents’ performance in open versus laparoscopic bench-model cholecystectomy in a hands-on surgical course. Int. J. Surg..

[13] Chmielecki D.K., Hagopian E.J., Kuo Y., Davis J.M. (2012). Laparoscopic cholecystectomy is the preferred approach in cirrhosis: A nationwide, population-based study. HPB.

[14] Schietroma M., Carlei F., Franchi L., Mazzotta C., Sozio A., Lygidakis N.J., Amicucci G. (2004). A comparison of serum interleukin-6 concentrations in patients treated by cholecystectomy via laparotomy or laparoscopy. Hepatogastroenterology.

[15] Buunen M., Gholghesaei M., Veldkamp R., Meijer D.W., Bonjer H.J., Bouvy N.D. (2004). Stress response to laparoscopic surgery: A review. Surg. Endosc..

[16] Targarona E.M., Pons M.J., Balagué C., Espert J.J., Moral A., Martínez J., Gaya J., Filella X., Rivera F., Ballesta A. (1996). Acute Phase is the Only Significantly Reduced Component of the Injury Response after Laparoscopic Cholecystectomy. World J. Surg..

[17] Watt D.G., Horgan P.G., McMillan D.C. (2015). Routine clinical markers of the magnitude of the systemic inflammatory response after elective operation: A systematic review. Surgery.

[18] Burugu H.R., Kandi V., Kutikuppala L.V.S., Suvvari T.K. (2020). Activities of Serum Ferritin and Treatment Outcomes Among COVID-19 Patients Treated with Vitamin C and Dexamethasone: An Uncontrolled Single-Center Observational Study. Cureus.

[19] Huang Y.-C.T., Bassett M.A., Levin D., Montilla T., Ghio A.J. (2006). Acute Phase Reaction in Healthy Volunteers After Bronchoscopy with Lavage. Chest.

[20] Arsalani-Zadeh R., Ullah S., Khan S., MacFie J. (2011). Oxidative Stress in Laparoscopic Versus Open Abdominal Surgery: A Systematic Review. J. Surg. Res..

[21] Chambers K., Ashraf M.A., Sharma S. (2020). Physiology, Hepcidin. StatPearls [Internet].

[22] Suuronen S., Kivivuori A., Tuimala J., Paajanen H. (2015). Bleeding complications in cholecystectomy: A register study of over 22,000 cholecystectomies in Finland. BMC Surg..

[23] Tandon A., Shahzad K., Nunes Q., Shrotri M., Lunevicius R. (2017). Routine preoperative blood group and save testing is Unnecessary for elective laparoscopic Cholecystectomy. J. Ayub Med. Coll. Abbottabad JAMC.

[24] Huilin L., Guihua L., Zhaoxing T. (2014). Changes in blood lymphocytes in sepsis patients. Zhonghua Wei Zhong Bing Ji Jiu Yi Xue.

[25] Ai S., Sun F., Liu Z., Yang Z., Wang J., Zhu Z., Du S., Guan W. (2018). Change in serum albumin level predicts short-term complications in patients with normal preoperative serum albumin after gastrectomy of gastric cancer. ANZ J. Surg..

[26] Komáromi A., Estenberg U., Hammarqvist F., Rooyackers O., Wernerman J., Norberg A. (2016). Simultaneous assessment of the synthesis rate and transcapillary escape rate of albumin in inflammation and surgery. Crit. Care.

